# Pediatric Neck Impalement Injuries: A Report of Two Cases and Review of the Literature

**DOI:** 10.7759/cureus.111516

**Published:** 2026-06-25

**Authors:** Ayaka Tsutsumi, Roxanna Hamidpour, Matthew Wagoner, Giovanni Ayala, Mehmet Erkilinc, Shin Miyata, Christopher Blewett

**Affiliations:** 1 Pediatric Surgery, SSM Health Cardinal Glennon Children's Hospital, St. Louis, USA; 2 General Surgery, Saint Louis University School of Medicine, St. Louis, USA; 3 Orthopedic Surgery, Saint Louis University School of Medicine, St. Louis, USA

**Keywords:** impalement injury, neck impalement injury, pediatric trauma, spine surgery, trauma

## Abstract

Penetrating neck impalement injuries in children are rare but potentially life-threatening. We present two cases to illustrate the clinical spectrum of this injury pattern and reinforce key management principles.

Case 1 was a 13-year-old female patient who sustained a wooden stick impalement to the right neck during a sledding accident. She was hemodynamically stable with a Glasgow Coma Scale (GCS) score of 15 and no neurological deficits. Computed tomography (CT) angiography showed a 2.5 cm soft tissue laceration with no vascular or aerodigestive injury. Case 2 was a 17-year-old female patient struck by a wire projectile from a lawnmower, presenting with immediate left upper extremity paresthesia but intact motor function and airway. CT confirmed a metallic foreign body traversing the spinal canal from T1-T2 to T2-T3 without vascular or aerodigestive injury.

Both patients underwent operative removal of a foreign body under general anesthesia. Case 1 required wound irrigation and suture repair; she was discharged on oral antibiotics without complications. Case 2 underwent wire extraction with intraoperative neurophysiological monitoring and received targeted antibiotic prophylaxis given agricultural contamination. She was discharged neurologically intact with a cervical collar and orthopedic follow-up.

These cases reinforce that pediatric neck impalement demands CT angiography prior to removal (if feasible), operative extraction in a controlled surgical setting, and vigilance for delayed complications, including carotid thrombosis, regardless of how benign the initial presentation appears.

## Introduction

Penetrating neck injuries in children are uncommon but carry significant morbidity and mortality due to the density of vital structures within this anatomical corridor, such as the carotid and vertebral arteries, jugular veins, trachea, esophagus, cervical spinal cord, and cranial nerve branches, all in close proximity with limited soft tissue buffering. The neck is classically divided into three zones as described by Roon and Christensen: Zone I extends from the clavicles to the cricoid cartilage, Zone II lies between the cricoid cartilage and the angle of the mandible, and Zone III extends from the angle of the mandible to the base of the skull [1]. Zone II, the most commonly injured region, is the zone involved in Case 1 of this report.

Children are particularly vulnerable to severe neck impalement injury for reasons beyond anatomy alone. Reduced subcutaneous fat, muscle mass, and connective tissue provide less mechanical protection to underlying vital structures [2], and the pediatric neck is proportionally shorter and more compact, positioning the internal carotid artery, which lies more medially in children than in adults, in closer proximity to the pharyngeal wall [3]. The most common mechanisms are accidental falls onto protruding fixed objects such as wooden stakes, metal rods, and bicycle handlebar components, accounting for up to 81% of pediatric impalement cases [2], with oral impalement from objects carried in the mouth representing a distinct and underappreciated mechanism predominantly affecting toddlers [3].

Foreign body impalement injuries present unique challenges distinct from high-velocity projectile trauma: the object may be tamponading active hemorrhage, its internal trajectory is often unpredictable, and the apparent benignity of the external wound frequently belies the severity of deeper injury. A fundamental diagnostic pitfall is that wound size is a poor predictor of internal injury extent. Toothbrush impalements have been shown to traverse deep into the mediastinum with no visible external evidence of severity [4], and oropharyngeal injuries can silently involve the internal carotid artery, with neurological deficits appearing hours to days later during a clinically deceptive lucid interval [3]. Cross-sectional imaging is therefore essential in the initial evaluation of all pediatric neck impalements.

We present two cases illustrating different clinical presentations, anatomical trajectories, and operative approaches and review key management principles applicable to this rare but consequential injury pattern.

## Case presentation

Case 1

History and Presentation

A 13-year-old female patient presented to our pediatric trauma center as a trauma major following a penetrating tree branch injury to the right neck sustained during a sledding accident. Emergency medical services (EMS) transported the patient to a pediatric trauma center for evaluation. 

Vitals on arrival and neurological status are summarized in Table 1. She endorsed right neck pain at the location of the penetrating injury (Figure 1). Physical examination was significant for a penetrating injury to the right neck with the foreign body, a wooden stick, still in situ within the wound. The wound was located in Zone II of the neck (between the cricoid cartilage and the angle of the mandible). There was no active hemorrhage, expanding hematoma, subcutaneous emphysema, stridor, hoarseness, or neurological deficits. The only other physical sign of injury was a superficial abrasion to the anterior right calf. Chest and pelvis X-rays in the trauma bay were negative for acute findings.

**Table 1:** Case 1 vital signs and neurological status on arrival bpm: beats per minute; GCS: Glasgow Coma Scale; SpO₂: oxygen saturation

Parameter	Patient value	Reference range
Heart rate	110 bpm	60-100 bpm
Blood pressure	141/82 mmHg	<120/80 mmHg
SpO₂	99% (room air)	95-100%
GCS score	15	15 (normal)
Neurological deficits	Absent	Absent

**Figure 1:**
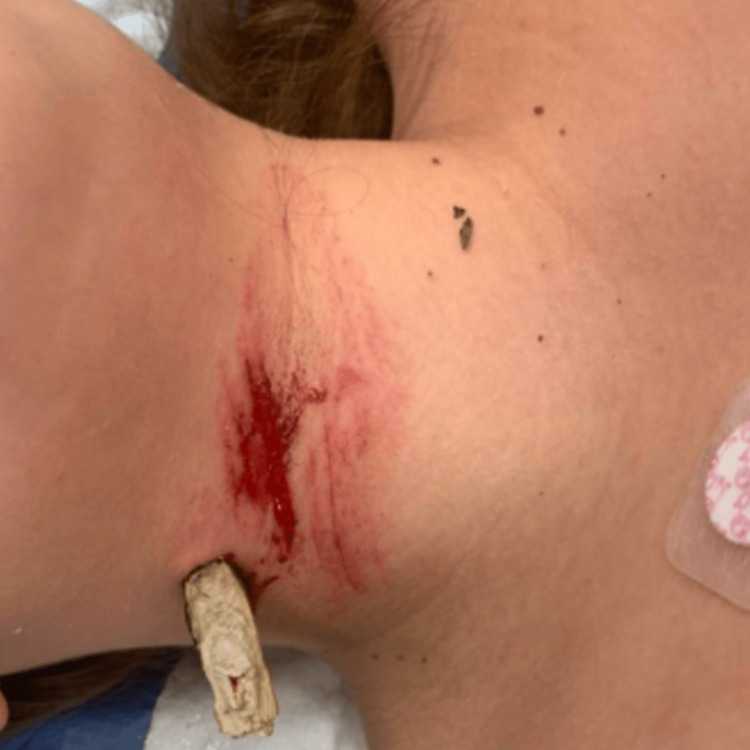
Clinical photograph upon arrival showing a right neck impalement injury

Imaging

Timely computed tomography angiography (CTA) of the neck was obtained, demonstrating a transversely oriented laceration of the right infrahyoid soft tissue with associated subcutaneous emphysema deep to the platysma but superficial to the sternocleidomastoid and strap muscles (Figure 2). The total length of the foreign body was about 6 cm, of which 2.5 cm penetrated the neck. The diameter was noted to be 1 cm on CTA. There was no evidence of vascular injury, contrast extravasation, or injury to adjacent aerodigestive structures.

**Figure 2:**
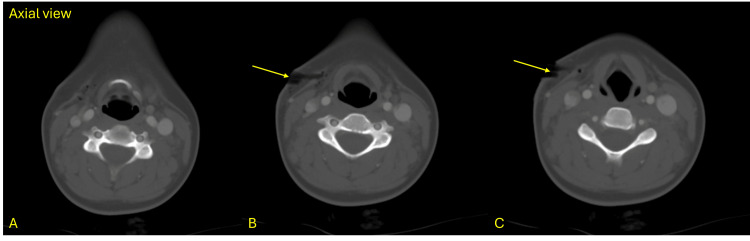
CTA imaging of the neck at the level of impalement injury (axial views) (A) Axial CTA image at the level of the cricoid cartilage without soft tissue windowing demonstrating the normal pre-injury anatomical reference level for comparison. (B) Axial CTA image at the level of the impalement injury demonstrating a transversely oriented soft tissue laceration in the right infrahyoid region (arrow). (C) Axial CTA image at a slightly more cephalad level demonstrating the superior extent of the soft tissue defect and associated subcutaneous gas tracking (arrow), with intact adjacent vascular structures bilaterally. CTA: computed tomography angiography

Operative Management

Operative intervention to remove the foreign body was pursued. The patient underwent induction of general anesthesia and intubation without complications. The wooden stick was removed manually without resistance, and there was no subsequent active hemorrhage. Following meticulous irrigation to confirm the absence of residual foreign body material, hemostasis was achieved, and the laceration was primarily repaired with interrupted absorbable suture.

Postoperative Course and Outcome

The patient was extubated without difficulty. She resumed oral intake postoperatively and was discharged home the same day. A five-day course of oral Augmentin was prescribed at discharge, consistent with previously documented management for penetrating injuries involving contaminated organic material [5]. The patient did not attend the scheduled follow-up appointment, and attempts to contact the patient's family by telephone were unsuccessful.

Case 2

History and Presentation

A 17-year-old female patient presented following a neck impalement with a haywire sustained during a lawn mowing accident. EMS was contacted and trimmed the wire on scene before transporting the patient to an outside hospital, where initial imaging was obtained prior to being transferred to the pediatric trauma center for further evaluation.

On arrival, vital signs and neurological status are summarized in Table 2. She reported left upper arm paresthesias and discomfort in the left neck (Figure 3). Physical examination was significant for a metal wire impalement in the left neck without other physical signs of trauma. There was no active hemorrhage, expanding hematoma, subcutaneous emphysema, stridor, hoarseness, or neurological deficits. No motor deficits were documented on initial examination.

**Table 2:** Case 2 vital signs and neurological status on arrival bpm: beats per minute; GCS: Glasgow Coma Scale; SpO₂: oxygen saturation

Parameter	Patient value	Reference range
Heart rate	106 bpm	60-100 bpm
Blood pressure	139/91 mmHg	<120/80 mmHg
SpO₂	99% (room air)	95-100%
GCS score	15	15 (normal)
Neurological deficits	Left upper arm paresthesias; no motor deficit	Absent

**Figure 3:**
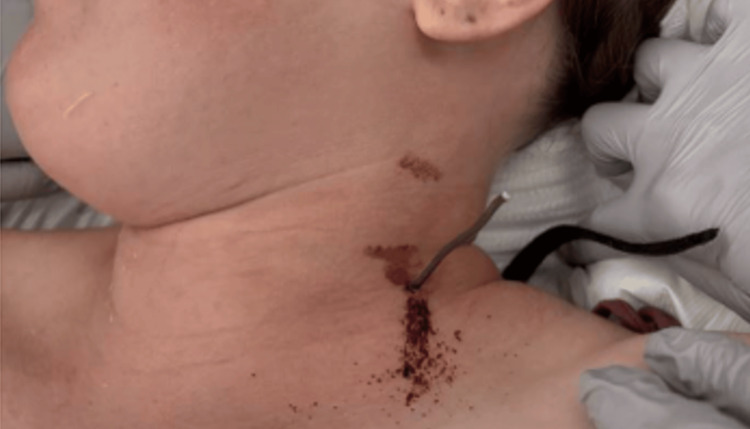
Clinical photograph upon arrival showing a left neck impalement injury

Imaging

Imaging performed at the outside hospital was reviewed. It included a non-contrast head CT without acute findings, cervical spine CT, and CTA of the neck and chest with findings of a metallic foreign body penetrating the spinal canal at T1-T2 with the tip terminating in the anterior spinal canal at T2-T3 and no major vascular injury, hematoma, or subcutaneous emphysema noted (Figure 4). No gross hemorrhage was identifiable within the spinal canal. The total length of the metallic object was about 15 cm, with a diameter of 5 mm, of which 9 cm penetrated the neck.

**Figure 4:**
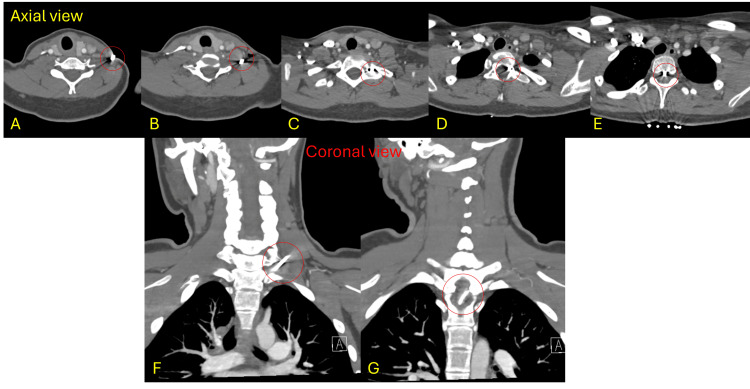
CTA demonstrating metallic foreign body trajectory (axial and coronal views) Axial views (A-E, top row): Sequential axial CTA images from cranial to caudal, each with the metallic wire highlighted by a red circle. Coronal views (F and G, bottom row): (F) Coronal CTA reconstruction demonstrating the full craniocaudal extent of the wire (red circle) from its lateral neck entry to the anterior spinal canal at T2-T3. (G) Coronal CTA reconstruction at a slightly more posterior plane, confirming the wire's trajectory through the neural foramen and its position within the spinal canal (red circle), without identifiable vertebral artery injury. CTA: computed tomography angiography

This case represents an exceptionally high-stakes anatomical trajectory with involvement of the spinal canal at T1-T2, placing the spinal cord at immediate risk of injury from movement of the wire. The absence of motor deficit despite cord-level penetration is consistent with rare reports in the literature of objects traversing critical neuroanatomical structures with minimal functional disruption, likely due to the narrow profile of the instrument and its trajectory through a relatively tolerant anatomical corridor [6].

Operative Management

A multidisciplinary operative team of orthopedic spine and trauma surgery was assembled for removal, an approach reflecting documented management of complex impalement injuries [5]. Intraoperative neurophysiological monitoring was employed throughout the case to provide real-time assessment of spinal cord function, a critical adjunct given the wire's anatomic position. After prepping the neck in a sterile fashion, the metallic wire was removed slowly, in a straight retrograde trajectory along its entry path, without lateral movement, consistent with published surgical principles for intracranial and intraspinal foreign body removal [5]. Neck compression was applied immediately after removal; there was no active hemorrhage or growing hematoma, and intraoperative neurophysiological monitoring remained at baseline throughout the procedure.

Postoperative Course and Outcome

The patient was admitted to the pediatric intensive care unit (PICU) postoperatively with spinal precautions overnight and hourly neurological checks. CT of the cervical spine without contrast, the following morning, confirmed the successful removal of the metallic foreign body, with no postoperative hematomas (Figure 5).

**Figure 5:**
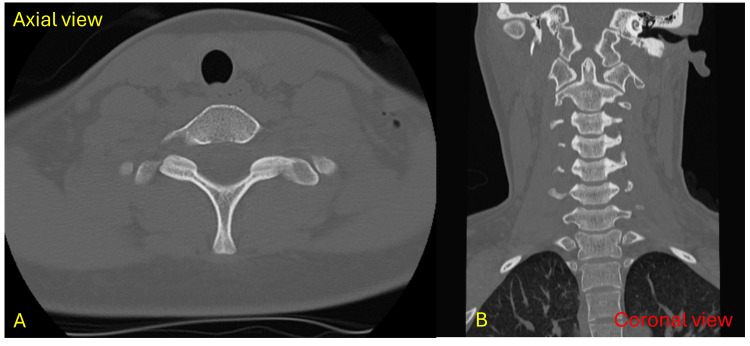
Postoperative CT of the cervical spine without contrast confirming complete metallic foreign body removal (A) Axial CT image at the T1-T2 level demonstrating the absence of any metallic foreign body within the spinal canal or paraspinal soft tissues. (B) Coronal CT reconstruction spanning the cervical and upper thoracic spine, confirming the complete removal of the wire along its entire prior trajectory, with no residual metallic artifact. CT: computed tomography

From an infectious disease standpoint, the patient received cefazolin for 24 hours as surgical prophylaxis. Given the agricultural origin of the wire and the attendant risk of soil-associated organisms, infectious disease specialists recommended an additional 72-hour course of metronidazole to provide anaerobic coverage [2]. This approach parallels the antibiotic strategy described in similar pediatric cases involving contaminated metallic foreign bodies [6]. Tetanus prophylaxis had been administered at the outside hospital.

The patient worked with physical and occupational therapy without limitations to mobility and was discharged home in a cervical collar with activity restrictions (avoiding sports, etc.).

## Discussion

These two cases illustrate the clinical spectrum of pediatric neck impalement injury, from a relatively superficial Zone II wooden stick impalement with a benign anatomical trajectory to a high-risk metallic wire impalement traversing the neural foramen and entering the spinal canal. Despite markedly different injury severity, both cases were managed with shared foundational principles: cross-sectional vascular imaging, controlled operative removal, multidisciplinary consultation, antibiotic prophylaxis, and structured postoperative surveillance.

Epidemiology and mechanism

Impalement injuries in children are rare, accounting for a small fraction of all pediatric trauma presentations. In a 10-year single-center review of pediatric impalement injuries, only 21 cases were identified among 4,826 total pediatric trauma presentations, with falls onto protruding fixed objects accounting for approximately 81% of cases [7]. The male predominance reported in most series was not apparent in our two cases, both of which involved female adolescents, a demographic reminder that impalement injuries are not exclusively a male or toddler phenomenon.

The anatomical region injured has major prognostic implications. In large cohort data, neck impalements represent a minority of cases but carry distinct risks related to the proximity of the carotid and vertebral arteries, jugular veins, trachea, esophagus, and cervical spinal cord [7].

The penetrating mechanism concentrates force on a small surface area, potentially enabling deep penetration at relatively low velocity, which is particularly relevant to wire projectile injuries, where the narrow cross-section of the object belies its penetrating capability.

Imaging strategy

For the above patients with protected airways and appropriate hemodynamics without any hard signs of vascular or aerodigestive injury on physical exam, cross-sectional imaging to further evaluate the trajectory of impalement is integral to operative planning. CT and CTA together provide the most comprehensive assessment, identifying the foreign body trajectory, characterizing the material (metallic vs. non-metallic), delineating proximity to the carotid and vertebral arteries, and screening for subclinical pneumothorax, pneumomediastinum, and mediastinal emphysema, a complication that may be present with no external signs of its severity [4].

CTA is specifically indicated when the injury trajectory is lateral and in anatomical proximity to the internal carotid artery. A literature review of 78 pediatric oropharyngeal and parapharyngeal impalement cases found that vascular injuries were detected in 47.4% of the cases reviewed and that left-sided injuries carried a significantly higher vascular injury rate than right-sided injuries (60.6% vs. 37.8%; p=0.046) [3]. Among patients sent home without imaging due to apparent symptom absence, all of them subsequently returned with severe complications, including neurological sequelae and death, a sobering reminder that the absence of symptoms at initial presentation does not exclude significant vascular injury [3].

For metallic foreign bodies within or near the spinal canal, CTA provides vascular mapping. However, this imaging modality may not be ideal in the presence of metallic streak artifacts. In such situations, digital subtraction angiography (DSA) is recommended to obtain a clear view and rule out vascular injuries [8]. Magnetic resonance imaging (MRI) is valuable for characterizing parenchymal injury in the spinal cord and posterior fossa and can be considered where the metallic composition of the foreign body can be excluded or confirmed as non-ferromagnetic, as it provides superior soft tissue resolution without ionizing radiation [6].

Operative principles

The single most critical principle in the field management of impalement injuries is that the impaled object must never be removed outside of a controlled surgical setting. The object may be tamponading active hemorrhage from adjacent vascular structures; premature removal risks converting a survivable injury into a rapidly fatal one [7]. In a pediatric bicycle handlebar impalement series, five of eight patients had the foreign body removed prior to hospital arrival, a practice the authors appropriately flagged as potentially dangerous despite the absence of major hemorrhage in those particular cases [9]. Public and first-responder education on this principle remains an important preventive priority.

In the operating room, extraction should be performed in a straight retrograde trajectory along the entry path, without lateral movement or rotational force, to minimize the risk of additional vascular or neural injury during removal [5]. This principle is especially critical when the foreign body is in proximity to the spinal cord or major vessels. For intraspinal foreign bodies, intraoperative neurophysiological monitoring is an invaluable adjunct, providing real-time feedback on cord function throughout the extraction.

For wooden or organic foreign bodies, complete removal requires meticulous intraoperative irrigation and direct visualization, as retained fragments are a major source of postoperative infection and abscess formation. Wood is radiolucent and may not be fully appreciable on pre- or postoperative imaging [10]. In one reported case, an intraoral wooden lollipop stick impalement led to a fulminant intraorbital abscess [11].

Carotid artery injury and the lucid interval

A critically important teaching point from the neck impalement literature is the lucid interval in internal carotid artery injury. Following oropharyngeal or parapharyngeal impalement, the internal carotid artery may sustain an intimal tear that initiates thrombosis. Because more than 50% of children have adequate collateral flow through the circle of Willis, neurological symptoms may be entirely absent at the time of initial evaluation, appearing only hours to days later as the thrombus propagates [3]. In a series of 78 pediatric cases, the mean interval from injury to neurological symptom onset was 32.5 hours, with a range of two hours to six days [3]. All four fatalities in that series involved left lateral soft palate injury, and all were associated with internal carotid artery thrombosis.

While neither of our patients showed signs of carotid injury on CTA, awareness of this complication is mandatory in any lateral neck impalement. Anticoagulation initiated before neurological symptom onset has been shown to reduce rates of thrombosis and stroke; once stroke has occurred, the benefit of anticoagulation is significantly diminished [3]. The threshold for CTA should be low for any lateral neck wound, and patients without imaging who are discharged must receive explicit return precautions for delayed neurological symptoms.

Aerodigestive complications

Penetrating neck wounds can also result in aerodigestive injury with subsequent pneumomediastinum and subcutaneous emphysema. These complications may present with tachycardia, fever, and crepitus in the neck, the last of which should always prompt immediate imaging. A recent case report described a two-year-old with a posterior pharyngeal wall defect from a pencil impalement who developed extensive retropharyngeal and mediastinal emphysema surrounding both internal carotid arteries, requiring operative repair under general anesthesia [12]. A one-year-old with a toothbrush injury developed mediastinal emphysema surrounding the left carotid arteries despite no visible wound on examination, with the only initial laboratory clue being an elevated C-reactive protein [4]. These cases underscore the principle that clinical examination is insufficient to exclude serious injury in pediatric neck and pharyngeal impalement; CT is the primary diagnostic tool.

## Conclusions

Pediatric neck impalement injuries are rare but potentially life-threatening events requiring systematic and multidisciplinary management. The two cases presented here illustrate the range of trajectories and severity possible within this injury category and the importance of consistent application of established trauma principles, regardless of limited external signs of injury. Cross-sectional imaging, preferably CTA, must be obtained before any attempt at foreign body removal. Impaled objects must not be extracted outside a surgical setting. Operative removal should proceed in a retrograde, straight-line fashion under direct visualization, with appropriate monitoring when the trajectory involves vascular or neurological structures. Antibiotic prophylaxis tailored to potential contaminating microorganisms, tetanus prophylaxis, and structured postoperative follow-up complete the management framework. Although neither case presented demonstrated carotid thrombosis with a lucid interval, the broader literature mandates awareness of this complication.
